# Genome-wide search and structural and functional analyses for late embryogenesis-abundant (LEA) gene family in poplar

**DOI:** 10.1186/s12870-021-02872-3

**Published:** 2021-02-24

**Authors:** Zihan Cheng, Xuemei Zhang, Wenjing Yao, Kai Zhao, Lin Liu, Gaofeng Fan, Boru Zhou, Tingbo Jiang

**Affiliations:** 1 State Key Laboratory of Tree Genetics and Breeding, Northeast Forestry University, Harbin, China; 2 Bamboo Research Institute, Nanjing Forestry University, 159 Longpan Road, Nanjing, 210037 China

**Keywords:** Poplar, LEA, Evolutionary analyses, Expression patterns, Salt stress

## Abstract

**Background:**

The Late Embryogenesis-Abundant (LEA) gene families, which play significant roles in regulation of tolerance to abiotic stresses, widely exist in higher plants. Poplar is a tree species that has important ecological and economic values. But systematic studies on the gene family have not been reported yet in poplar.

**Results:**

On the basis of genome-wide search, we identified 88 LEA genes from *Populus trichocarpa* and renamed them as *PtrLEA*. The *PtrLEA* genes have fewer introns, and their promoters contain more cis-regulatory elements related to abiotic stress tolerance. Our results from comparative genomics indicated that the *PtrLEA* genes are conserved and homologous to related genes in other species, such as *Eucalyptus robusta*, *Solanum lycopersicum* and *Arabidopsis*. Using RNA-Seq data collected from poplar under two conditions (with and without salt treatment), we detected 24, 22 and 19 differentially expressed genes (DEGs) in roots, stems and leaves, respectively. Then we performed spatiotemporal expression analysis of the four up-regulated DEGs shared by the tissues, constructed gene co-expression-based networks, and investigated gene function annotations.

**Conclusion:**

Lines of evidence indicated that the *PtrLEA* genes play significant roles in poplar growth and development, as well as in responses to salt stress.

**Supplementary Information:**

The online version contains supplementary material available at 10.1186/s12870-021-02872-3.

## Background

Abiotic stresses, such as high salt, drought, and low temperature, challenge plant growth and development, resulting in decreased production and quality [1]. In evolution, however, plants have developed a serial of mechanisms at molecular, physiological, and biochemical levels, in order to minimize the effects from the abiotic stresses [2]. For example, transcription factors and protein kinases can regulate downstream signal transduction pathways, which eventually lead to physiological responses to the stresses [3]. On the other hand, functional proteins in plants, such as the late embryogenesis abundant (LEA) proteins can eliminate cellular content of active oxygen species, in order to protect macromolecular substances and alleviate damages caused by the abiotic stresses [4].

The first LEA protein was discovered in cotton [5]. Researchers found that the protein was substantially accumulated during the dehydration and maturation period of cotton seeds, in order to protect the seeds from damages [5]. Afterwards, more LEA proteins have been identified in *Arabidopsis* [6], rice [7], barley [8], and other species. The LEA proteins are mainly located in cytoplasm, mitochondria, and nucleus of plants, even lightly located in the endoplasmic reticulum [9]. According to conserved domains of the LEA proteins, they can be divided into eight clusters, including LEA_1, LEA_2, LEA_3, LEA_4, LEA_5, LEA_6, dehydrin, and seed maturation protein (SMP) [10]. Nevertheless, there exist discrepancies in classification between different species [9].

When plants are subjected to challenging environment, such as high salt and drought, ion concentration of the cell sap will rise rapidly, causing irreversible damages to the cells [11, 12]. The LEA proteins function as the responsive in assisting plants to tolerate such stresses as dehydration [10]. These proteins contain a high proportion of glycine, lysine and histidine, but lack alanine and serine [13]. Therefore, the LEA proteins, especially those in the first cluster, have high hydrophilicity and thermal stability, redirecting water molecules in cells, binding salt ions, and eliminating the active oxygen free radicals accumulated in cells due to dehydration [14]. Furthermore, the LEA proteins can prevent collapse of cell structure by binding to cell membrane [15]. In addition, they can combine with misfolded proteins through molecular chaperones and repair the misassembled proteins to restore their biological activity [16].

Increasing lines of evidence indicated that the LEA proteins play important roles in plant responses to abiotic stresses. Over-expression of *OsEml* gene increased ABA sensitivity and osmotic tolerance in rice, when challenged with drought stress [17]. Similarly, transgenic peppers over-expressing *CaLEA1* gene were able to enhance the sealing of stomata, and increase expression of drought and salt stresses responsive genes [18]. In addition, *Arabidopsis AtLEA14* [19] and *Setaria italica SiLEA14* [20] genes have been reported to confer salt tolerance. Recently, *TaLEA* in wheat [21], *ZmLEA3* in corn [22], and *SmLEA* in *Salvia miltiorrhiza* [23] were also reported to have functions in stress tolerance. However, studies in poplar are lacking.

In this study, we identified 88 LEA genes in poplars, and we analyzed the evolutionary relationships, gene duplication events, cis-acting sequences of LEA family members. In addition, we systematically analyzed the structure and function of LEA genes, especially the expression of poplar LEA genes in different tissues with and without salt stress. This research provides new information of poplar LEA family genes, and provides reference value of the gene evolution and function.

## Results

### Identification and characterization of the LEA genes in poplar

**Fig. 1:**
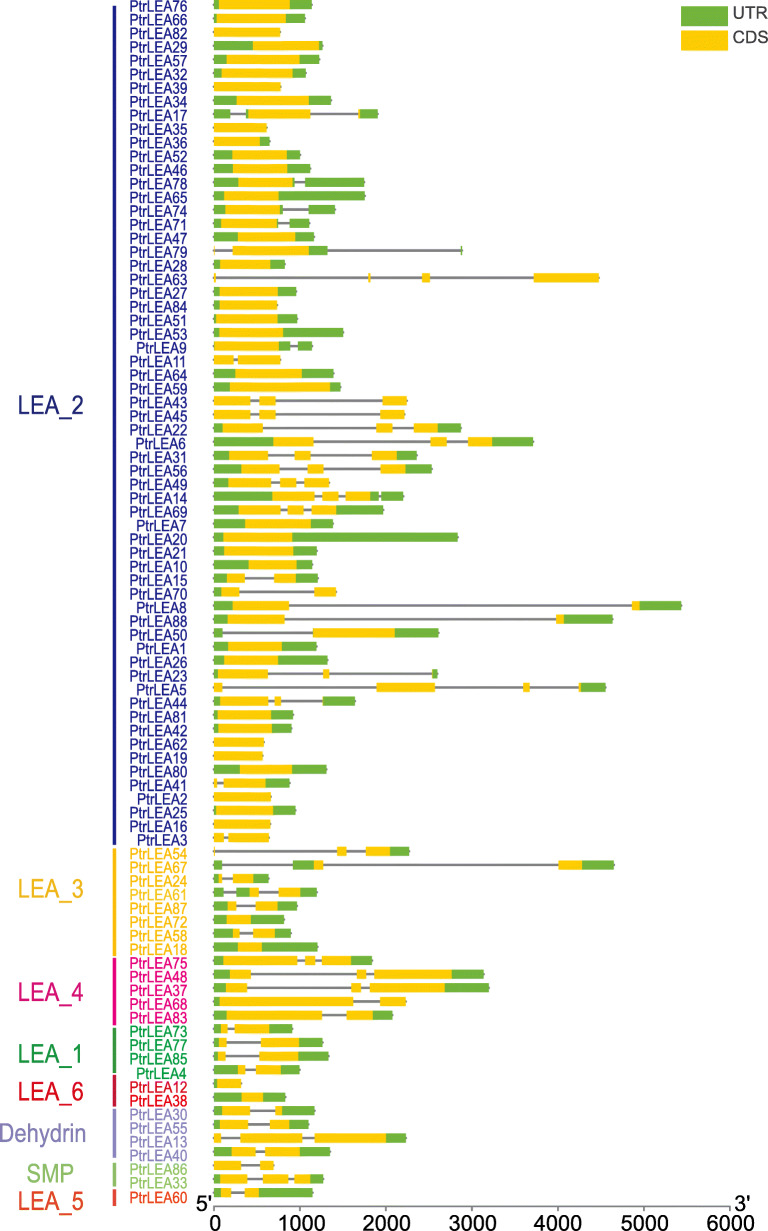
Structure of the *PtrLEA* genes in poplar. The gene names on the left are arranged according to the phylogenetic tree. The right represents the gene structure. The green boxes represent untranslated regions, the yellow boxes denote coding regions, and the lines indicate introns

A total of new 88 *LEA* genes were identified from *Populus trichocarpa*. Since these genes are different from previous studies [24], we renamed each of them as *PtrLEA*, followed by a number according to its localization on the poplar genome. We then classified the *PtrLEA* genes into eight clusters with various numbers of genes each (Additional file 1: Table S1). It appears that cluster LEA_2 has the maximal number of genes (60), followed by cluster LEA_3 (8), cluster LEA_6 (5), clusters LEA_1 and the Dehydrin (4 genes each), clusters LEA_4 and SMP (2 genes each), and LEA_5 cluster (1). In general, genes in the same cluster share similar structure. Structural characteristics of introns and exons of the *PtrLEA* genes are given in Fig. 1. We found that the number of introns per gene ranges from 0 to 3, of which 39 genes have no introns, 26 genes contain one intron, 20 genes harbor 2 introns, and only 3 genes have 3 introns.

Proteins encoded by the 88 *PtrLEA* genes displayed varied physicochemical properties (see additional file 1: Table S1). Their molecular lengths and weights fall into the range of 82–616 amino acids and 9.018–66.913 kDa, respectively. The values of theoretical isoelectric point (pI) range from 4.6 to 10.38. Regarding the grand average of hydropathicity, we found that 25 of the proteins have indices greater than 0, which are treated as hydrophobic proteins. In contrast, 61 proteins have indices less than 0, which are hydrophilic proteins. The values of aliphatic index are between 31.25 and 118.06. The protein instability coefficients run the gamut from 12.44 and 68.11, of which two-thirds of the proteins have values less than 40.

### Phylogenetic analyses of the LEA genes in both poplar and Arabidopsis

**Fig. 2:**
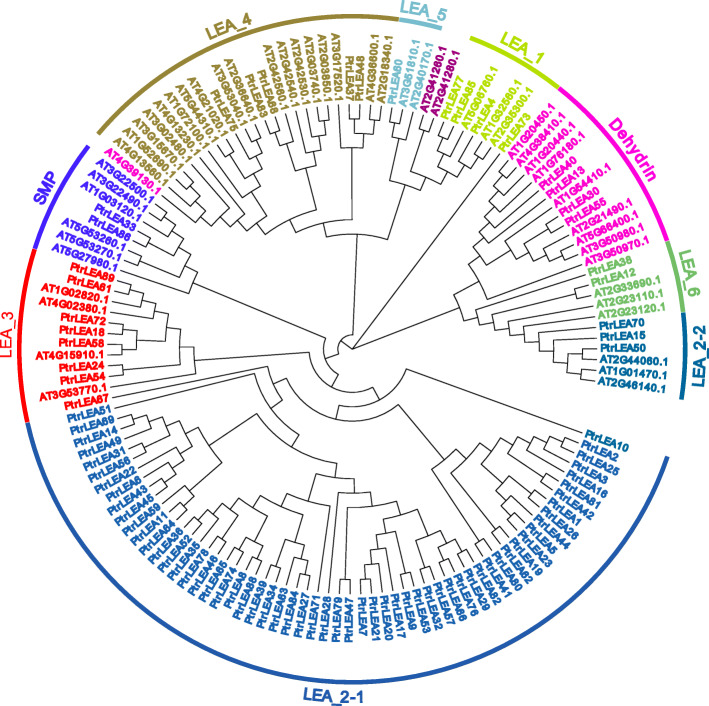
Phylogenetic analysis of LEA proteins in poplar and Arabidopsis. A. total of 139 LEA proteins were subject to multiple sequence alignment, and a phylogenetic tree was constructed by use of MEGA7 with the maximum likelihood method and1000 bootstrap resampling. Each color represents one group

In order to better understand evolutionary relationships of the LEA gene family from both poplar and Arabidopsis, we classify the genes based on similarity of their protein sequences [25]. A phylogenetic tree is shown in Fig. 2. In general, cluster LEA_2 can be divided into two sub-clusters, namely LEA_2–1 and LEA_2–2. In contrast, clusters LEA_1, LEA_4, and LEA_5 can be grouped into larger clades. Similarly, clusters Dehydrin, LEA_6, and LEA_2–2 form other clades.

In addition, we built a phylogenetic tree using only the 88 *PtrLEA* genes. Similarly, they can be divided into 8 clusters that belong to two major clades; that is, cluster LEA_2 and part of cluster LEA_3 in one clade, and the rest of the genes in another clade (Additional file 2: Fig. S1).

### Chromosomal distribution of the *PtrLEA* genes and cross-species collinearity analysis

**Fig. 3:**
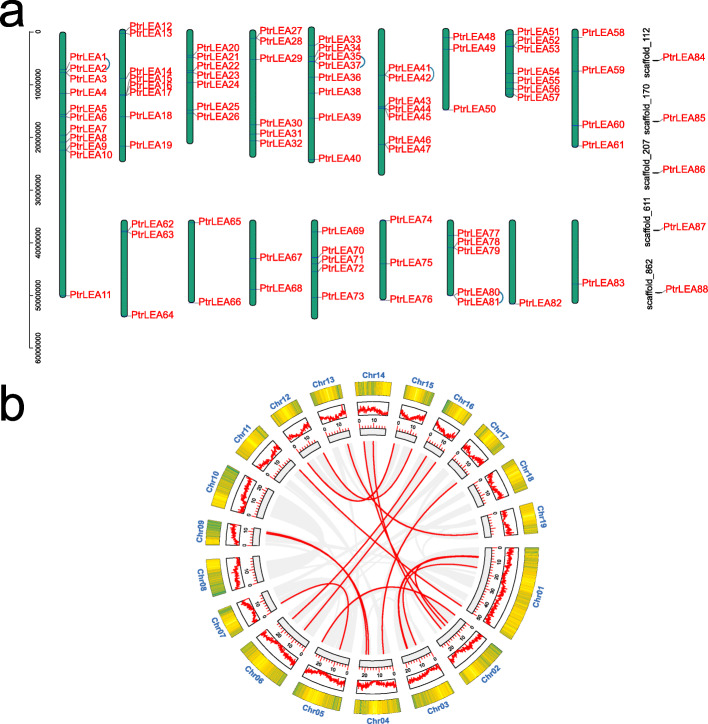
Chromosomal localization of the *PtrLEA* genes and gene duplication events. **a** Distribution of the genes on chromosomes of poplar, the small blue are represents pairs of tandem repeated genes. **b** Duplicate pairs of the *PtrLEA* genes in poplar. The red lines represent collinear pairs of the *PtrLEA* genes, while the gray lines indicate collinear pairs of all poplar genes

Genomic distribution of the 88 *PtrLEA* genes is varied by chromosomes or scaffolds (Fig. 3). Chromosome 1 harbors the maximal number of genes (11). In contrast, only one gene is located on chromosome 17 and chromosome 18 respectively. In addition, five *PtrLEA* genes are distributed on different scaffolds. Interesting, no *PtrLEA* genes were found on chromosomes 8 and 19.

Within-genome duplication events of the 88 *PtrLEA* genes were analyzed by use of the MCscan [26]. We found that eight genes on respective chromosomes 1, 5, 6 and 16 display four pairs of tandem duplication events (Fig. 3a). In addition, we also found that 37 genes exhibit 19 pairs of fragment duplication events uniformly distributed on the corresponding chromosomes (Additional file 3: Table S2, Fig. 3b).

**Fig. 4:**
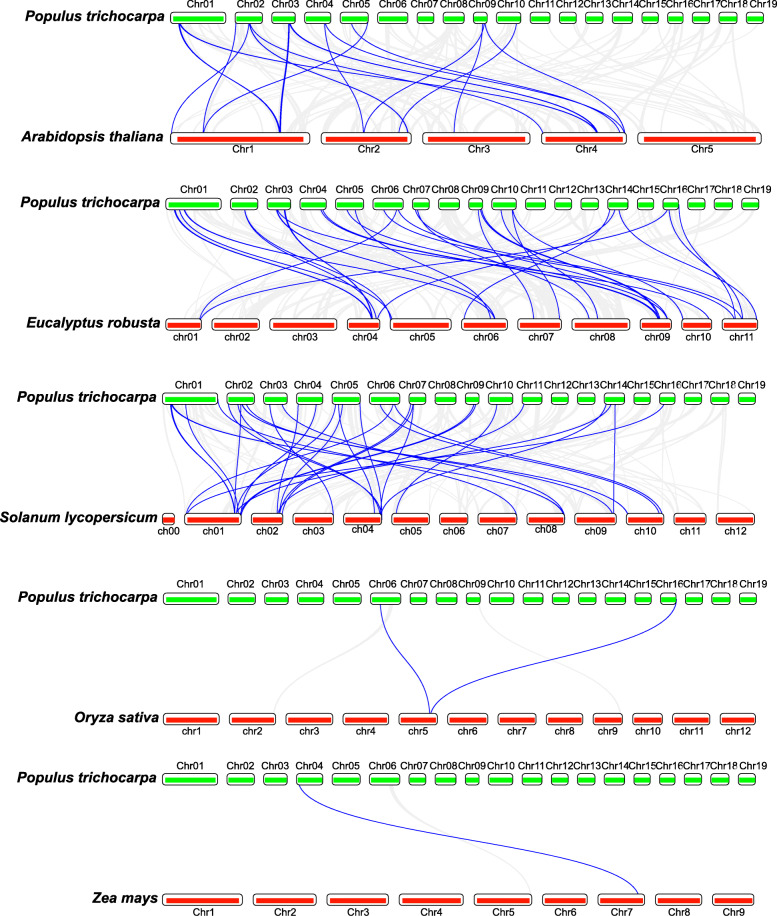
Collinearity map of the *PtrLEA* genes in poplar to other five species. The blue lines denote collinearity between the *PtrLEA* genes and other species, while the gray lines represent collinearity between the poplar genome and other species

We further compared DNA sequence similarity of the *PtrLEA* genes to the related genes from other species. We constructed collinearity maps of *Populus trichocarpa* along with three dicotyledons (*Eucalyptus robusta*, *Solanum lycopersicum* and *Arabidopsis*) and two monocotyledons (*Zea mays* and *Oryza sativa*), respectively. As shown in Fig. 4, we identified 32 repetitive events in *Eucalyptus robusta*, 34 in *Solanum lycopersicum*, 21 in *Arabidopsis*, 1 in *Zea mays,* and 2 in *Oryza sativa* (Additional file 4: Table S3). We found that the collinearity blocks were mainly distributed on the first five chromosomes of poplar. Several *PtrLEA* genes, such as *PtrLEA16*, *PtrLEA30* and *PtrLEA55*, have orthologous genes in *Eucalyptus robusta*, *Arabidopsis* and *Solanum lycopersicum*. Similarly, other genes, such as *PtrLEA80,* have orthologs in *Oryza sativa* and *Solanum lycopersicum*.

The ratio of Ka/Ks represents the percentage of non-synonymous substitution rate (Ka) over synonymous substitution rate (Ks) for a pair of protein-coding genes. It is an important reference for species selection evolution. The ratio > 1 indicates positive selection; the ratio = 1 denotes neutral evolution; and the the ratio < 1 means negative selection [27]. As shown in additional file 3: Table S2, the ratios of the *PtrLEA* genes fall into the range of 0.11 to 1.05, with an average value of 0.43. Only one pair of duplication events has a ratio greater than 1, while the remaining duplicative gene pairs have ratios less than 1. These suggest that the *PtrLEA* genes have been subjected to purification selection in the process of evolution. According to the divergence rate of 1.5 × 10–8 synonymous replacement rate site per year [28], we predicted that the divergence time of the *PtrLEA* genes repetitive events to be approximately from 4.72 to 106.72 million years ago (MYA), with an average of 25 MYA.

### Cis-elements analysis in promoters of the the *PtrLEA* genes

**Fig. 5:**
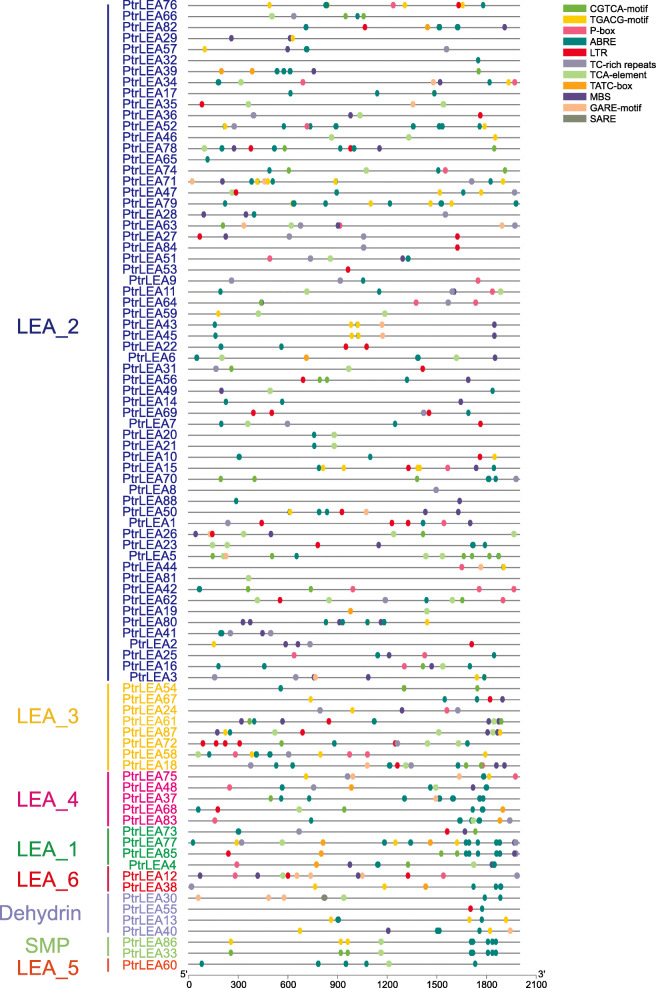
Predicted of cis-elements in the promoters of the P*trLEA* genes. The gene names on the left are arranged according to the phylogenetic tree. The patterns in different colors on the right represent different cis-elements

We extracted upstream 2000-bp sequences from each of the *PtrLEA* genes, followed by cis-element prediction using the PlantCARE (http://bioinformatics.psb.ugent.be/webtools/plantcare/html/) (Additional file 5: Table S4). We found that many elements are related to regulation of abiotic stresses (Fig. 5), such as the MBS elements for drought stress [29], the LTR elements for low temperature stress [30], and the TC-rich repeats elements for plant protection and stress stimulation [31]. Interesting, among the hormone-related elements (P-box, ABRE, GARE-motif, CGTCA-motif, TGACG-motif, SARE), the ABRE element is present in the promoters of all of the *PtrLEA* genes. This element is mainly involved in abiotic stress regulation in response to the ABA signaling pathway [32]. Given the evidence that such elements relevant to abiotic stress are abundantly present in the promoter regions of the *PtrLEA* genes, these genes may play significant roles in regulation of abiotic stress responses in poplar.

### *PtrLEA* gene expression analysis across tissues without salt stress

**Fig. 6:**
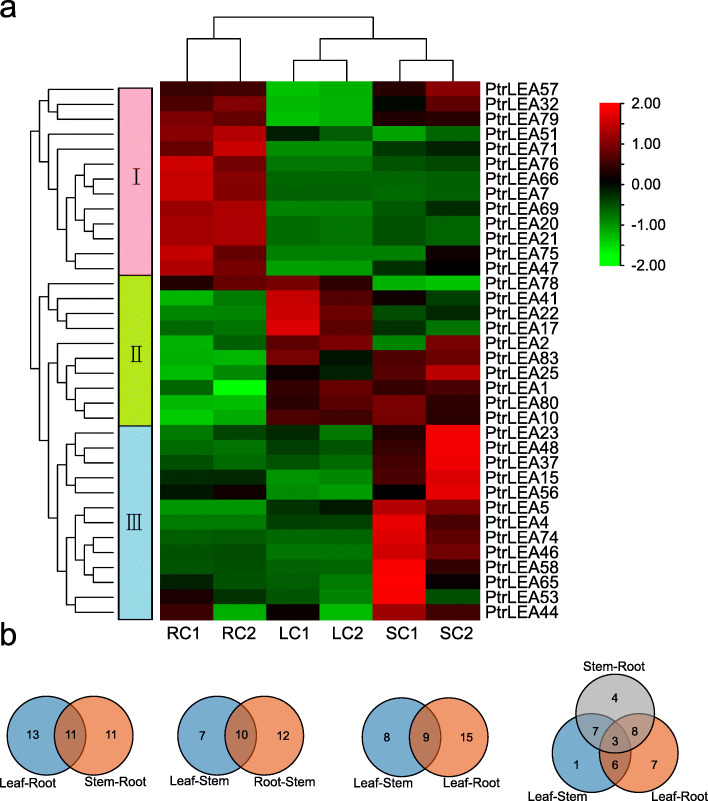
Tissue specific expression analysis of the *PtrLEA* genes without salt stress. **a** Heatmap of differentially expressed genes (DEGs) between tissues. Cluster analyses were based on log2FPKM. Red represents highly expressed genes and green represents low expressed genes. The left side represents gene clusters. RC, LC, and SC are stands for untreated roots, leaves, and stems, respectively. **b** Venn diagram of DEGs between tissues without salt stress

To characterize tissue-specific gene expression patterns of the *PtrLEA* genes, we compared their expression across three tissues (root, stem, and leaf) by RNA-Seq data (Additional file 6 Table S5). The genes can be divided into three groups that are highly expressed in roots, leaves, and stems, respectively (Fig. 6a). Through pairwise tissue comparisons, we identified 22 DEGs in root-stem, 17 DEGs in stem-leaf, and 24 DEGs in root-leaf (Fig. 6b). We then identified shared DEGs between two such comparisons with one common tissue (Fig. 6b). A total of 10 shared genes were found in the comparisons of root-stem and stem-leaf, 11 such genes of root-stem and root-leaf, and 9 such genes of stem-leaf and root-leaf. Finally, we identified 5 genes that are shared in all of the comparisons. The fold-changes of gene expression are shown in Additional file 7: Table S6.

### *PtrLEA* gene expression analysis under salt stress

**Fig. 7:**
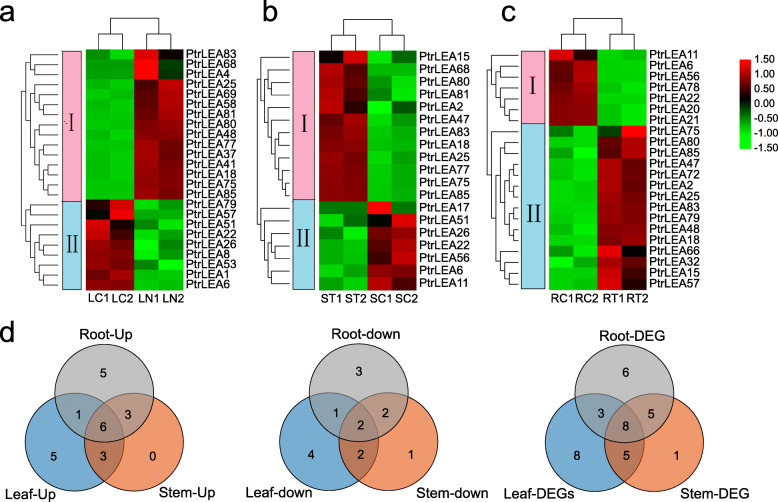
Analysis of differentially expressed genes (DEGs) under salt stress. **a** Heatmap of DEGs in leaves under salt stress. LC and LT stand for untreated and treated leaves, respectively. **b** Heatmap of DEGs in stems under salt stress. SC and ST denote untreated and treated stems, respectively. **c** Heatmap of DEGs in roots under salt stress. RC and RT indicate untreated and treated roots, respectively. **d** Venn diagram of DEGs between different tissues under salt stress

We explored expression patterns of the *PtrLEA* genes across different tissues under salt stress, using RNA-Seq data (Additional file 6 Table S5). Statistical evidence from the heatmap clearly indicated that the genes can be divided two groups (Fig. 7a). One group represents genes that are up-regulated in leaves and down-regulated in roots. The other group of genes displays an opposite pattern (Fig. 7a). The fold-changes of gene expression are shown in Additional file 8: Table S7.

Identification of DEGs that are responsive to salt stress can help shed light on gene functions. We identified 24 DEGs in leaves, including 15 and 9 up- and down-regulated genes (URGs and DRGs), respectively. Similarly, 22 DEGs (15 URGs and 7 DRGs) were found in roots, followed by 19 DEGs (12 and 7) in stems. The numbers of URGs are greater than that of DRGs across the three tissues. According to the magnitude of gene expression changes in response to salt stress, *PtrLEA85* displayed the maximum fold-change in roots (7.25X). In contrast, *PtrLEA56* represented the maximal down-regulation (− 5.85X) in the same tissue. In leaves, *PtrLEA75* had the maximal fold-change (10.73X), the opposite was *PtrLEA6* (− 2.92X). In stems, similar gene pairs were *PtrLEA68* (8.10X) and *PtrLEA17 (*− 6.47X). In addition, we analyzed shared DEGs between tissues (Fig. 7d). We identified 13 shared genes in the root-stem pair, 11 such genes in root-leaf, and 13 such genes in leaf-stem. Only 8 DEGs were shared across the three tissues.

### Verification of *PtrLEA* genes expression by RT-qPCR

**Fig. 8:**
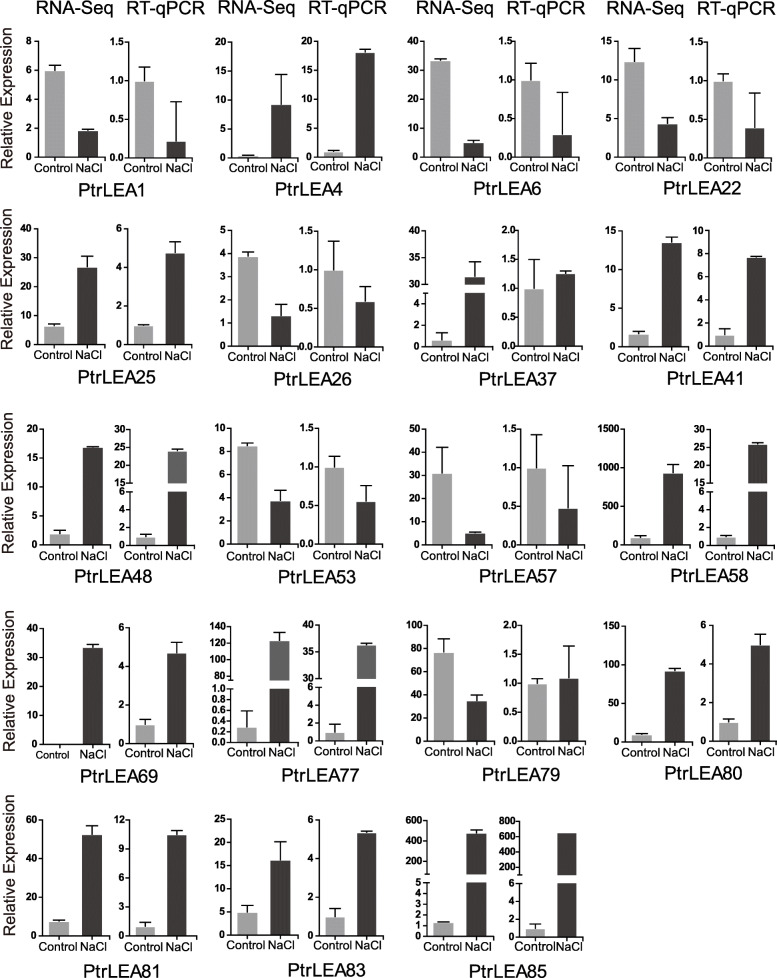
DEG expression levels in leaves from both RNA-Seq and RT-qPCR platforms. The expression in RNA-Seq was quantified by fragments per kilo-bases per million mapped reads (FPKM)

In order to verify the accuracy of the RNA-Seq data, we performed RT-qPCR analyses on all of the DEGs identified from each tissue. In general, the results from RT-qPCR and RNA-Seq are consistent, with a few exceptions (Fig. 8). In leaves, expression data of *PtrLEA37* and *PtrLEA57* differ between the two platforms (Fig. 8). In stems, similar discrepancy was observed for *PtrLEA56* (Additional file 9: Fig. S2). In roots, discrepancies were found for *PtrLEA11* and *PtrLEA20* (Additional file 10: Fig. S3). These deserve further investigation.

### Tempo-spatial expression pattern of the *PtrLEA* genes in response to salt stress

**Fig. 9:**
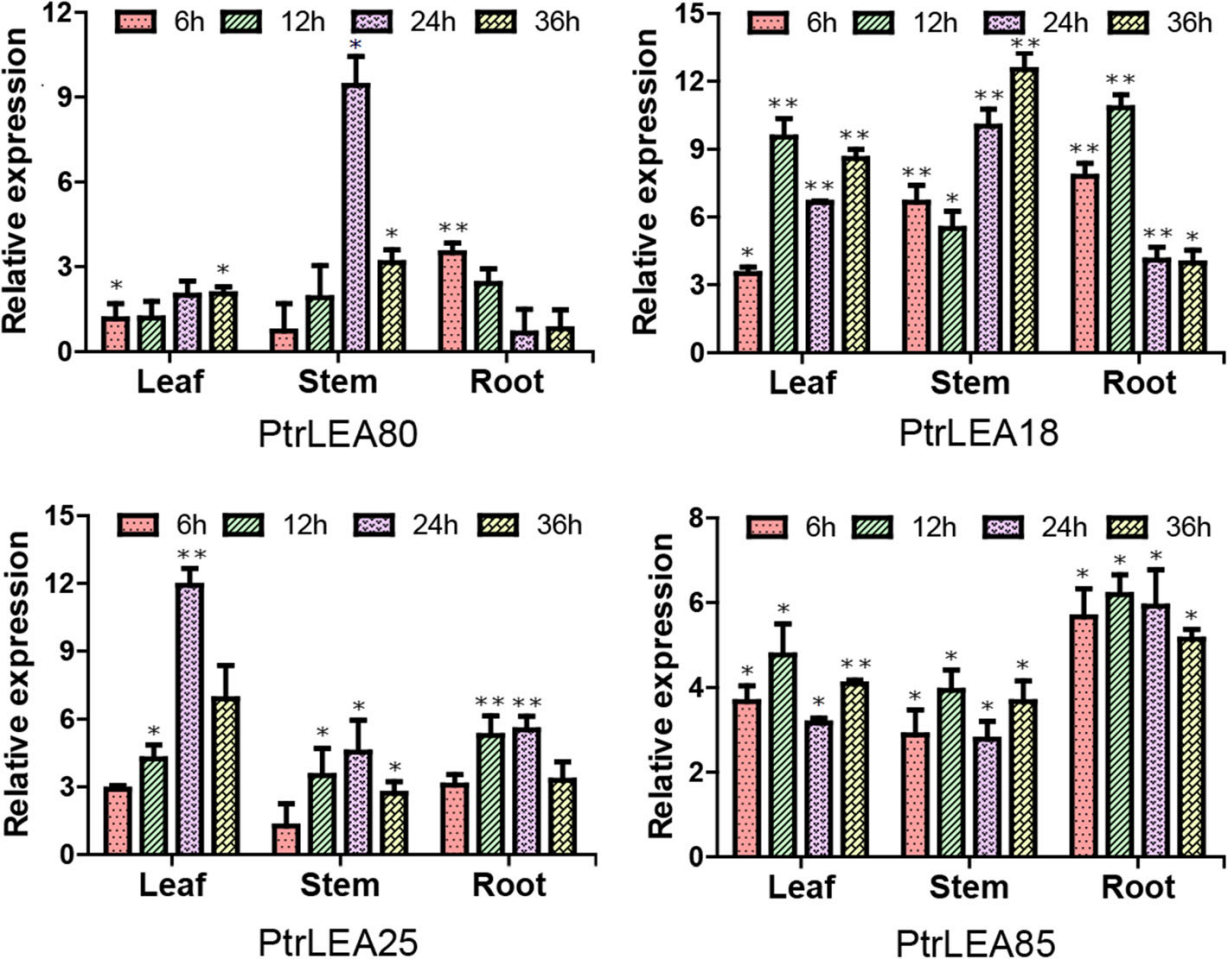
Tempospatial expression analysis of the 4 up-regulated genes under salt stress (150 mM). RT-qPCR analysis was performed with respective samples from roots, stems and leaves at each time point. The data was homogenized by log function. The data was processed using the 2-ΔΔCt method. Asterisks indicate significant differences between with and without salt stress (t test, *P < 0.05, ** *P* < 0.01)

Plants often respond to abiotic stresses by increasing the expression of stress-resistant genes [33]. In order to further explore dynamic gene expression patterns in response to salt stress across the tissues, we selected four significantly up-regulated DEGs that are shared by the tissues. Using RT-qPCR, we explored tempospatial expression patterns of the four genes. In general, four genes represented relatively high expression levels across the time course and over the three tissues. In leaves, both *PtrLEA85* and *PtrLEA18* had a peak at 12 h, while *PtrLEA25* gene reached a peak at 24 h (Fig. 9). In stems, similar results were observed for *PtrLEA80* and *PtrLEA25* genes (24 h), in contrast to *PtrLEA18* gene (12 h) (Fig. 9). In roots, the contrasting genes were between *PtrLEA18* (12 h) and *PtrLEA80* (6 h) (Fig. 9). These lines of evidence indicated that the genes displayed different expression patterns by tissues. In the same tissue different genes exhibit similar or unique responses to the salt stress.

### Gene co-expression and gene ontology analysis

**Fig. 10:**
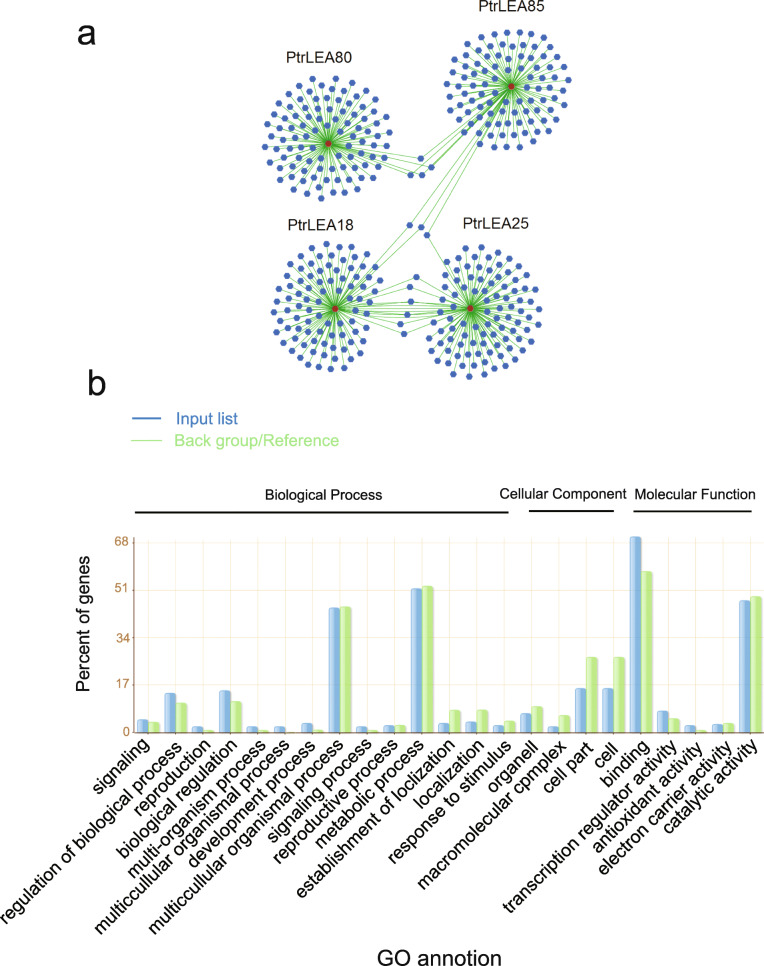
Co-expression-based gene networks. **a** Gene networks of the four *PtrLEA* genes. The red center dot represents each of the four *PtrLEA* genes. The surrounding blue dots indicate genes with highly collected expression patterns. **b** GO analysis of all selected genes (top 100 from each network)

Using Spearman correlation, we identified genes that are significantly co-expressed with the four up-regulated genes mentioned above. The top 100 such genes were selected for functional annotation (Additional file 11: Table S8). As shown in Fig. 10a, the centers of the four gene networks correspond to the four *PtrLEA* genes. Interesting, some genes are shared across the networks. For example, the *PtrLEA80* network has four shared genes with the *PtrLEA85* network. The *PtrLEA18* and *PtrLEA25* networks share seven genes (*Potri.005G072100.1*, *Potri.014G070200.1*, *Potri.T070900.1*, *Potri.019G037800.1*, *Potri.003G13340.1*, *Potri.012G092000.1* and *Potri.011G149300.1*). Similarly, two genes (*Potri.001G150400.1* and *Potri.001G309100.1*) are shared by the *PtrLEA85* and *PtrLEA18* networks. Only one gene (*Potri.017G094500.1*) is shared by the *PtrLEA85* and *PtrLEA25* networks. The share genes might reflect similar features in gene regulations in response to salt stress.

Using the agriGO online software (http://systemsbiology.cau.edu.cn/agriGOv2/index.php), we perform gene set enrichment analysis, based on the genes selected. Results are showed in Fig. 10b. We found that many *PtrLEA* genes are involved in various biological processes, such as biological regulation and response to stimulus. Regarding molecular functions, the *PtrLEA* genes are enriched in the antioxidant activity that is related to abiotic stress.

## Discussion

The LEA gene family widely exists and plays important roles in plants, especially in regulation of growth and development under abiotic stresses [34]. In this study, we identified 88 LEA genes from the poplar genome, which can be divided into eight clusters. Regarding the numbers of genes and clusters, our results are different from those of previous studies [24]. The discrepancies might be attributed to the improvement of plant genome annotation, and more genes being identified and classified in the present study as well. We found that the LEA_2 cluster is the largest one. Similar findings were observed in tea [35], *Sorghum bicolor* [36] and wheat [37]. This change may be attributed to the improvement of plant genome annotation, and more LEA genes being identified in the present study by different classification methods. This cluster can be further divided into two sub-clusters, which is similar to the studies on upland cotton [38]. We guessed that the poplar genome had undergone whole-genome replication events during the evolution process, and the genome has undergone multiple chromosomal rearrangements and fusions, which has promoted the amplification of many gene families [39, 40].

The shrinkage of introns in the *PtrLEA* genes may impact the time from transcription to translation, which may promote the rapid expression of genes in response to environmental changes [41]. We found the poplar LEA gene family has relatively few introns. Interesting, as many as 44% of the family members contain no introns, 53% of the genes harbor 0–1 introns, and only 3% of the genes have 3 introns. These results are similar to those from previous studies [42].

Mechanisms of gene family evolution include DNA fragment duplication, tandem duplication, and conversion events [43]. Duplication events may lead to the emergence of new genes, which contribute to the diversity of gene functions so that plants can improve adaptation to challenging environment [44]. In the present study, we found that both tandem duplication and fragment duplication events exist in the *PtrLEA* gene family. Fragment duplication events (19 pairs) are far more frequent than tandem duplication events (4 pairs), suggesting that the former might be the main force in promoting amplification of the *PtrLEA* genes. In addition, we analyzed the ka/ks ratios of the repeated gene pairs, the majority of which are less than 1. Therefore, we suspected that the *PtrLEA* genes have undergone purification selection over the evolutionary process.

In order to explore the evolutionary relationship of the LEA gene family across different species (5), we selected both dicotyledonous (3) and monocotyledonous (2) plants for comparisons. We found that the *ptrLEA* genes share the best homology with those from the eucalyptus, and the worst with those from rice. In addition, we found that the *ptrLEA* genes have more collinearity gene pairs with the dicotyledonous plants, compared to the monocotyledonous plants.

Cis-elements in promoter regions play significant roles in gene regulation and expression. Thus investigation of cis-elements can help identification of genes related to specific functions, such as stress resistance and plant developments. Many known such elements have been reported to be involved in plant stress responses, such as MBS, ABRE, P-box, LTR, and TGACG-motif. We found that the ABRE element is present in the promoters of all of the *PtrLEA* genes, and the other elements occur in some of the genes. These results are similar to previous studies [37]. This line of evidence suggests that the *PtrLEA* genes may regulate responses to abiotic stress in poplar.

Using RNA-Seq data, we found that expression of the *PtrLEA* genes varied significantly across different tissues without salt treatment. Evidence from comparative genomics and functional annotations indicate that the *PtrLEA* genes play important roles in regulation of poplar growth and development. For example, *AT2G46140*, which is homologous to *PtrLEA15*, plays an important role in regulation of primary and secondary metabolites [45]. Both *AT1G02820* (homologous to *PtrLEA58*) and *AT2G46300* (homologous to *PtrLEA17*) impact pollen germination and tube growth [46]. *AT1G32560* (homologous to *PtrLEA4*) is involved in the phytochrome A-signaling pathway [47].

Under salt stress, plants will activated certain signaling pathways, in order to induce corresponding cellular responses [12]. The inducibility of corresponding genes by the stress can be interpreted in the way that these genes might be involved in the pathways. In this study, we identified DEGs in each tissue by contrasting salt treatment. We detected 24 such genes in roots, 19 genes in stems, and 23 genes in leafs. Among these genes, eight are shared across the tissues. Interesting, two (*PtrLEA79* and *PtrLEA57*) of the eight genes were found to display opposite expression patterns across the tissues. We then suspected that the two genes play complex functions in regulation of poplar growth and development and responses to salt stress. Evidence from gene function annotations indicated that the eight shared genes are involved in regulation of complex functions. For example, *AT5G06760.1* (homologous to *PtrLEA85*) is involved in carbohydrate metabolism contributing to Arabidopsis growth and development [48]. Arabidopsis gene *AT2G36640.1 (*homologous to *PtrLEA75)* is able to interact with *bHLH109* and enhance tolerance of cells to emergency challenges [49]. Both *AT5G06760.1* (homologous to *PtrLEA85*) and *AT3G54200.1* (homologous to *PtrLEA80*) play important roles in regulation of the ABA signaling pathway in Arabidopsis [50, 51]. *AT4G02380.1* (homologous to *PtrLEA18*) is related to plant freezing tolerance in Arabidopsis [52]. Using four of the shared up-regulated genes, we analyzed temporal and spatial gene expression patterns. We found that these genes displayed different patterns by time course and by tissues, and in the same tissue different genes exhibit similar or unique responses to the salt stress, which reveals complex responses in the regulatory network of plant abiotic stress processes.

If some genes always have similar expression changes in a physiological process or in different tissues, then we have reason to believe that these genes are functionally related. In this study, we constructed gene co-expression networks, focusing on the four shared genes mentioned above. In fact, the four gene networks are cross-linked to one another through shared genes in each network, suggesting complex regulations of the *PtrLEA* genes in response to salt stress. We then analyzed the co-expressed genes for functional annotation. We identified enriched GO terms related to abiotic stresses, such as biological regulation, response to stimulus, and antioxidant activity. Evidence from comparative genomics indicated that many genes in the networks are related to both plant development and responses to abiotic stresses. For example, *AT4G35090.1* (ortholog of *Potri.008G109100.1)* regulate plant roots growth and salt stress tolerance [53], and it also regulate leaf senescence and shedding of floral organs by removal of reactive oxygen species [54]. Similarly, *HSL1*, which is orthologous to *Potri.016G136500.1*, is involved in seed maturation [55]. *HSP101* (homologous to *Potri.015G056900.1*) regulates response to heat stress [56]. These lines of evidences indicate that the *PtrLEA* genes may play central roles in the regulation of these complex biological functions.

Under salt stress, plants can produce a series of regulatory mechanisms, such as osmotic balance, antioxidant system, and ROS scavenging mechanisms [57]. These mechanisms interact with one another to improve plant stress tolerance. A large number of abiotic stress-related genes were involved in the mechanisms, including the LEA proteins [34], glycosyltransferases [58], and ROS scaling genes [59]. These genes can regulate the disorder of physiological metabolism caused by the abiotic stresses. LEA genes are widely involved in abiotic stress and play important roles in improving plant stress tolerance [34]. In this study, we analyzed mRNA expression of the *PtrLEA* genes. However, further instigation into mechanistic understanding of the genes is deserved. In addition, there are still a large number of stress-related genes to be mined. It is promising to develop salt tolerant poplar plants by selecting combinatory central genes for genetic engineering.

## Conclusions

In this study, we performed systematic studies on the LEA gene family in poplar. We identified 88 *PtrLEA* genes and divided them into 8 clusters, according to their protein sequence similarities. These genes are distributed on 16 chromosomes of poplar. Using RNA-Seq data that was collected from two conditions (with and without salt stress), we detected 24, 22 and 19 DEGs in roots, stems and leaves, respectively. Then we performed tempospetial expression analysis of the four up-regulated genes shared by the tissues, followed by development of gene coexpression-based networks and functional annotations. These lines of evidence indicated that the *PtrLEA* genes play important roles in regulation of poplar growth and development, as well as of responses to salt stress.

## Methods

### Identification of LEA proteins in *Populus trichocarpa*

The genome-wide data of *Populus trichocarpa* was obtained from Phtozome online website (https://phytozome.jgi.doe.gov/pz/portal.html), and the typical LEA protein domains (PF03760, PF03168, PF03242, PF02987, PF0477, PF10714, PF0492, and PF00257) were downloaded from the Pfam database (http://pfam.xfam.org/). Potential poplar LEA proteins were scanned against the poplar genome by use of the HMMER3.0 program [60], followed by manual verification with the SMART database (http://smart.embl-heidelberg.de/) and the PFAM database (http://pfam.xfam.org/). Proteins without the LEA domains were removed. Molecular mass and isoelectric points of each PtrLEA protein were predicted by use of the ExPASy website (http://web.expasy.org/protparam/).

### Gene structure and phylogenetic tree analysis of the LEA proteins

The poplar genomic sequences were obtained from the Phytozome database (https://phytozome.jgi.doe.gov/pz/portal.html). The coding sequence and genomic sequence of each LEA gene were aligned to analyze gene structure, followed by visualization with the TBtools software [61]. LEA Protein sequences of both *Populus trichocarpa* and *Arabidopsis* were downloaded from the Phytozome database and TARE databases (https://www.arabidopsis.org/), respectively. Using full-length protein sequences, we performed multiple sequence alignment with the ClustalW [62]. Phylogenetic analysis was conducted by use of the MEGA7 software, including the maximum likelihood method with bootstrap analysis for 1000 repetitions [63].

### Chromosome location and gene duplication of the poplar LEA genes (*PtrLEA*)

DNA sequences from the LEA gene family were mapped onto the genome of *Populus trichocarpa*. Distribution of the genes on the chromosomes or scaffolds was calculated by the TBtools software [61]. Duplicate events of the *PtrLEA* genes were calculated using the MCscan pairs [26]. In addition, we used the Dual Synteny Plotter [61] to analyze the collinearity between the the *PtrLEA* and the homologous genes from other species (*Eucalyptus robusta*, *Solanum lycopersicum*, *Arabidopsis*, *Zea mays* and *Oryza sativa*), followed by visualization with the TBtools software [61]. We used the KaKs_Calculator software [64] to calculate the ratio of non-synonymous substitution and synonymous substitution (Ka/Ks) for duplication gene pairs. We also applied the methods of Koch [65] to calculate the divergence time of each gene pair.

### Cis-acting element analysis

For each *PtrLEA* gene, the 2000-bp DNA sequence upstream of the start codon was obtained from the Phytozome v12.1 database (https://phytozome.jgi.doe.gov/pz/portal.html). We then used the online tool PlantCRAE (http://bioinformatics.psb.ugent.be/webtools/plantcare/html/) to extract cis-acting elements that will be visualized by use of the TBtools software [61].

### Plant material and stress treatments

The plant material used in this study was di-haploid *Populus simonii × Populus nigra*, which was planted in the experimental field of Northeast Forestry University, Harbin, China. The seedlings were grown in bottles with 1/2 MS culture medium in the greenhouse at 25 °C and 16/8-h light/dark cycles. For salt stress, one-month-old poplar seedlings were treated with 150 mM salt for 0 h, 6 h, 12 h, 24 h, and 36 h, respectively. Then samples of respective roots, stems and leaves were collected at each time point and treated with liquid nitrogen, followed by storage in the − 80 °C refrigerator. All treatments had three biological replicates.

### Analysis and validation of RNA-Seq

Using RNA-Seq, **w**e explored gene expression patterns of the *PtrLEA* gene family across different tissues under salt stress. The data was described in our previous studies [66]. We used DESeq2 [67] to identified differentially expressed genes (DEGs) with two standards, including absolute log2 (fold change) > =1 and adjusted *p*-value <= 0.05. To verify the accuracy of RNA-Seq data, we also performed RT-qPCR analysis on the differential expression genes. For details, please refer to our previous studies [68]. The primer sequences are given in Additional file 12: Table S9.

### Gene co-expression analysis and gene ontology annotation

We selected interesting genes for co-expression-based gene network analysis. We used the Spearman correlation coefficients to selected relevant genes from the RNA-Seq data [66]. Gene selection was based on *P*-value > 0.05. We then focused on the top 100 genes for network analyses using Cytoscape software [69] for visualization. Gene ontology (GO)-based function annotations were performed by use of the agriGO v2.0 [70].

## Supplementary Information

**Additional file 1: Table S1.** The list of 88 LEA genes identified in this article.

**Additional file 2: Figure S1.** Phylogenetic analysis of poplar LEA protein.

**Additional file 3: Table S2.** The list of 23 pairs repetitive events in poplar LEA genes and its Ka/Ks ratio.

**Additional file 4: Table S3.** The homologous relationships between the poplar LEA genes and the other species.

**Additional file 5: Table S4.** The list of cis-regulatoty elements of *PtrLEA* genes promoter**.**

**Additional file 6: Table S5.** Expression data of *PtrLEA* genes in three different tissues under salt and without salt stress.

**Additional file 7: Table S6.** The fold-changes of differentially expressed *PtrLEA* genes across different tissues without salt stress.

**Additional file 8: Table S7.** The fold-changes of differentially expressed *PtrLEA* genes under salt stress.

**Additional file 9: Figure S2**. DGE levels of RNA-Seq and RT-qPCR in stems.

**Additional file 10: Figure S3.** DGE levels of RNA-Seq and RT-qPCR in roots.

**Additional file 11: Table S8.** The top 100 correlation genes of genes expression of *PtrLEA85*, *PtrLEA18*, *PtrLEA25* and *PtrLEA80.*

**Additional file 12: Table S9.** The primers sequences used in this study.

## Data Availability

All data generated or analyzed during this study are included in this published article and its supplementary information files. The raw sequencing data used during the study have been deposited in NCBI SRA with the accession number SRP267437.

## Abbreviations

LEA: Late Embryogenesis-Abundant
MYA: Million years ago
DEGs: Differentially expressed genes
SMP: Seed maturation protein
Ka: Non-synonymous substitution rate
Ks: Synonymous substitution rate
URGs and DRGs: Up- and down-regulated genes
GO: Gene ontology
FPKM: Fragments per kilo-bases per million mapped reads
