# Supplementary figures and images for: Genome-wide search and structural and functional analyses for late embryogenesis-abundant (LEA) gene family in poplar
Source: BMC Plant Biol. 2021 Feb 24;21:110. doi: 10.1186/s12870-021-02872-3 (PMC7903804; doi:10.1186/s12870-021-02872-3)

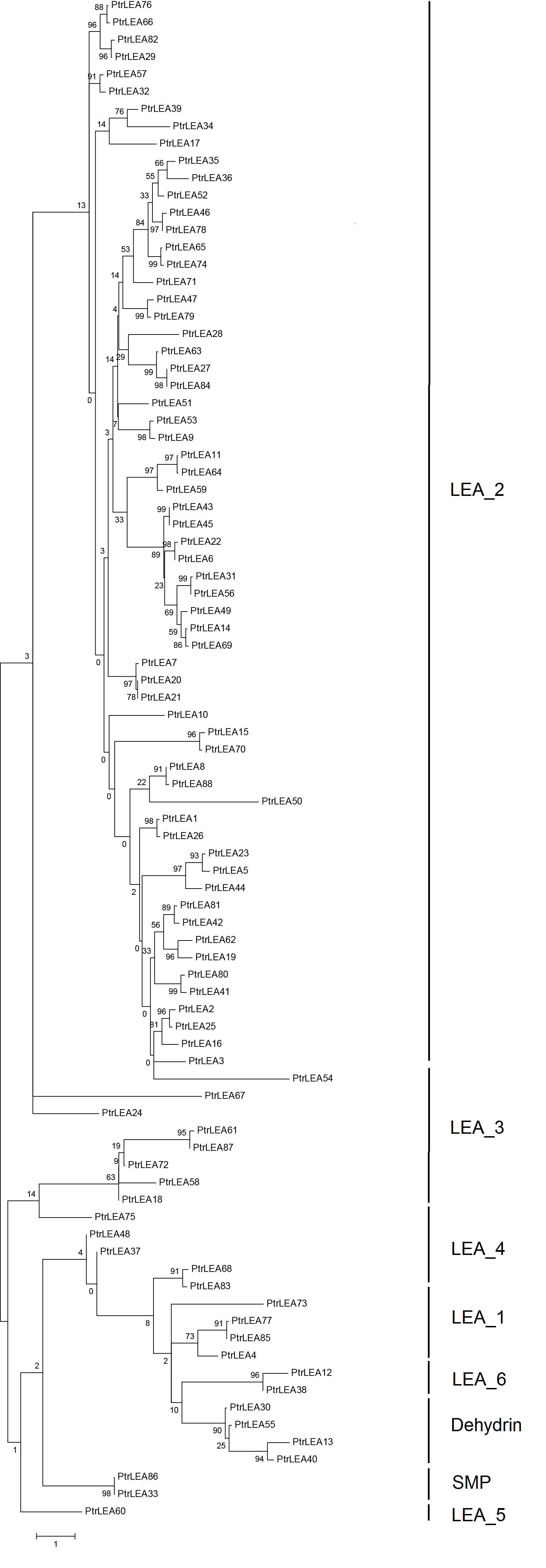

Supplement: Supplementary file 2 — Additional file 2: Figure S1. Phylogenetic analysis of poplar LEA protein. [file 12870_2021_2872_MOESM2_ESM.png]

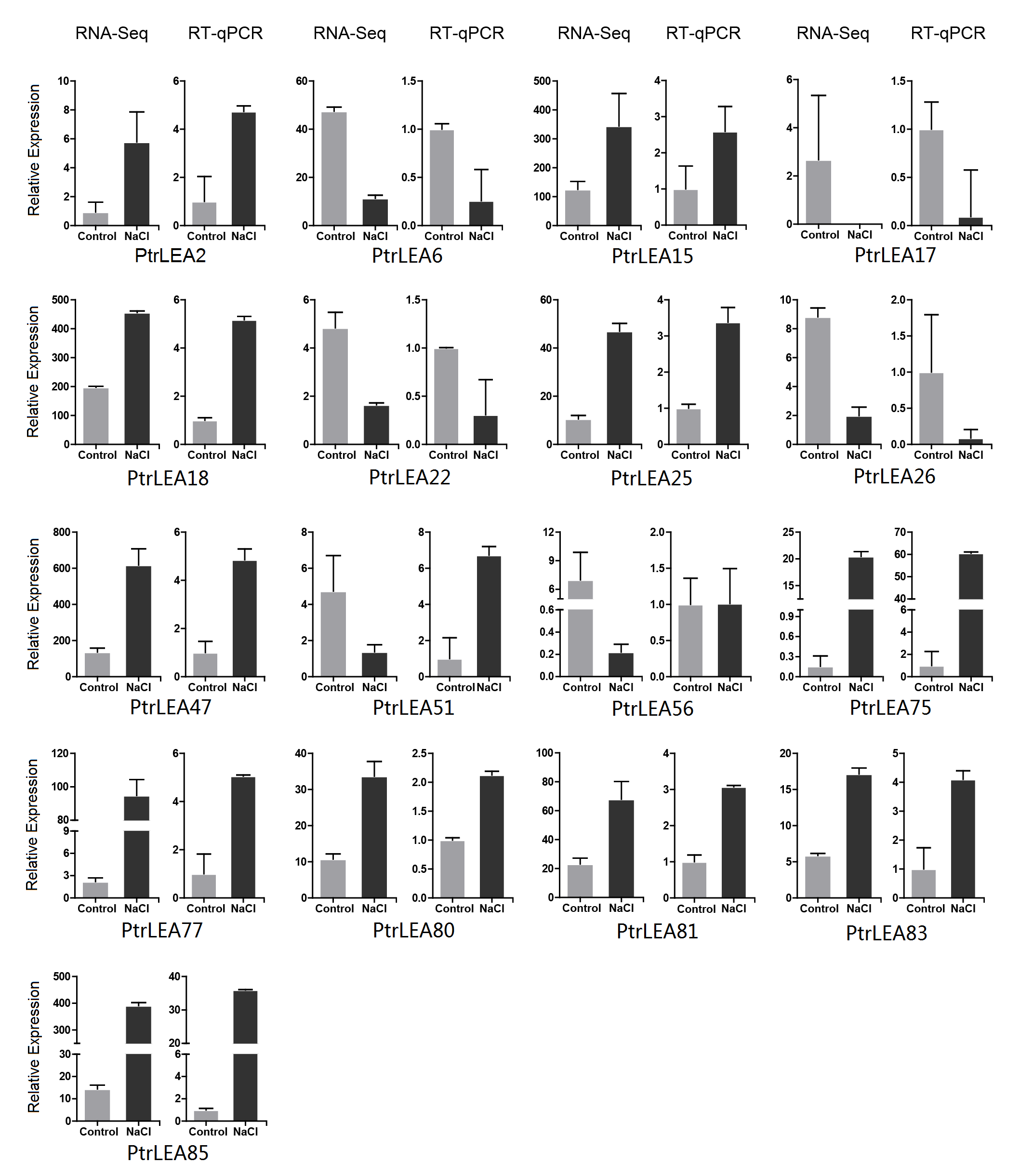

Supplement: Supplementary file 9 — Additional file 9: Figure S2. DGE levels of RNA-Seq and RT-qPCR in stems. [file 12870_2021_2872_MOESM9_ESM.png]

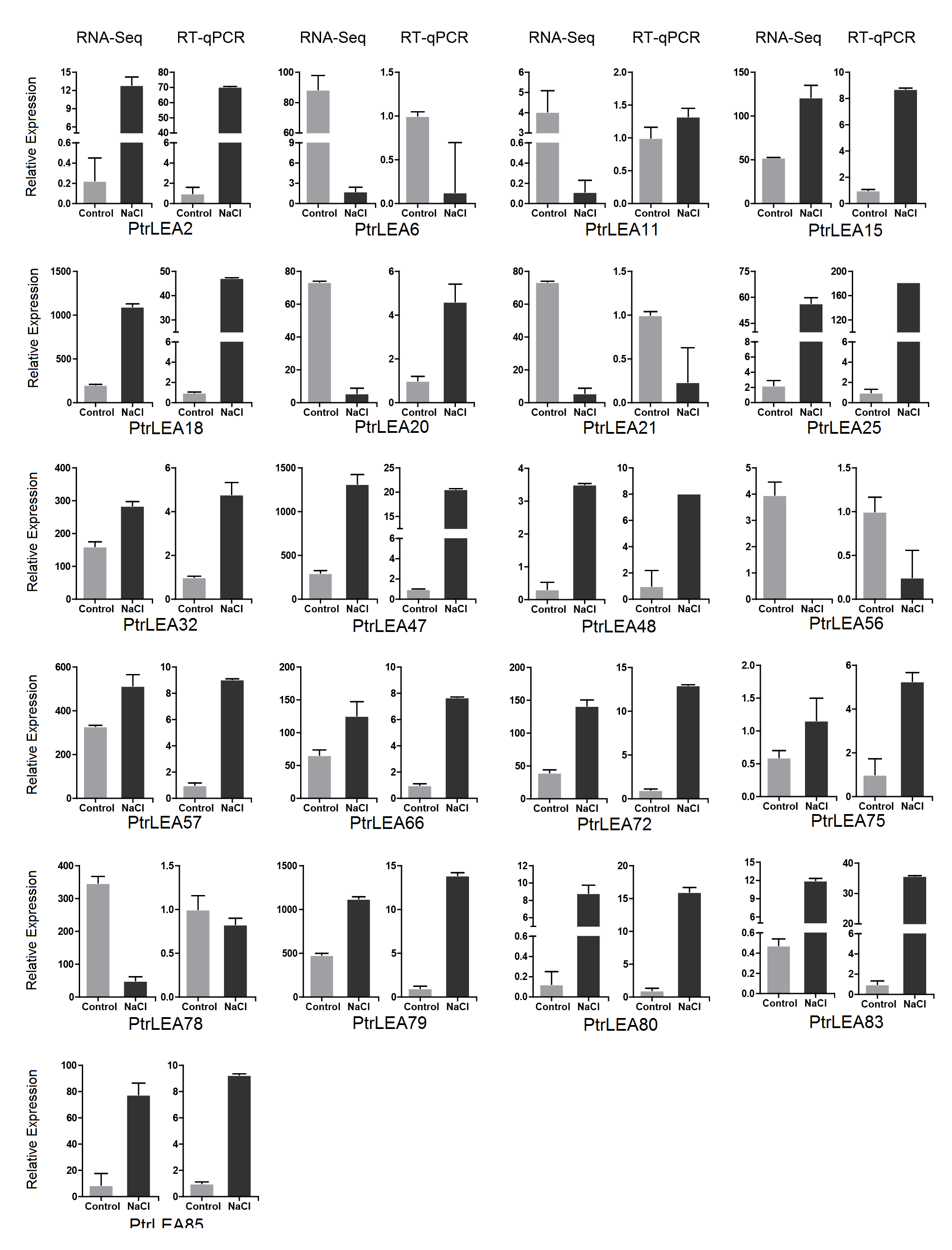

Supplement: Supplementary file 10 — Additional file 10: Figure S3. DGE levels of RNA-Seq and RT-qPCR in roots. [file 12870_2021_2872_MOESM10_ESM.png]
